# Associations between Self-Perceived and Desired Health-Related Physical Fitness in Spanish Children

**DOI:** 10.3390/children9091314

**Published:** 2022-08-29

**Authors:** Laura Redondo-Gutiérrez, Rocío Carballo Afonso, Antonio Molina, Miguel A. Sanchez-Lastra, Carlos Ayán

**Affiliations:** 1 Facaultad de Ciencias de la Educación y el Deporte, Universidad de Vigo, 36005 Pontevedra, Spain; 2 Departamento de Didácticas Especiais, Facultade de Ciencias da Educación e do Deporte, Universidade de Vigo, Campus A Xunqueira s/n, 36005 Pontevedra, Spain; 3 Faculty of Health Sciences, The Research Group in Gene—Environment and Health Interactions (GIIGAS), Institut of Biomedicine (IBIOMED), University of Leon, Campus de Vegazana s/n, 24071 León, Spain; 4 Instituto de Investigación Sanitaria Galicia Sur (IIS Galicia Sur) Sergas-UVIGO, 36312 Vigo, Spain

**Keywords:** health perception, physical activity levels, physical fitness, self-concept, children

## Abstract

Health-related physical fitness (HRPF) has emerged as an important marker of health among children. It is usually defined as a multidimensional construct consisting of cardiorespiratory endurance; muscular strength and endurance; and flexibility. Currently, health policies are aimed at getting children to increase their HRPF levels. Understanding the relationship between the perceived and the desired levels of fitness can be important to avoid the abandoning of physical activity practice. We analyzed the associations between the self-perceived and the desired health-related physical fitness. A modified version of the self-perceived HRPF questionnaire was completed by 330 children (148 girls, mean age: 10.78 ± 0.67 years, and 182 boys, mean age: 10.82 ± 0.61 years). Fitness was measured through tests selected from the Eurofit battery. The questions regarding desired HRPF showed moderate internal consistency (Cronbach’s α: 0.671) and good test–retest reliability (ICC: 0.761). In general, children shared a desire to improve their self-perceived HRPF. Those who perceived themselves as already being fit were the ones who showed the strongest desire for achieving higher HRPF levels.

## 1. Introduction

The increasing prevalence of overweight and obesity during childhood and adolescence is a global public health concern due to its relationship with health problems, especially in the adulthood [1]. On the contrary, being physically fit improves health outcomes and helps to achieve and maintain an adequate body composition, influencing the general quality of life [2,3].

In fact, it has been reported that low cardiorespiratory and musculoskeletal fitness in adolescence are significantly associated with all-cause mortality later in life [4]. Physical education classes are considered of great importance to increase both physical activity levels and fitness, given the evidence that they represent a critical period for both the acquisition of motor skills and the practicing of physical activity [5,6]. However, strong evidence shows that physical fitness (especially cardiorespiratory fitness) of children and adolescents has declined globally over the past few decades [7].

Health has been defined for more than half a decade by the World Health Organization [8] as not only the absence of disease and infirmity but a global welfare state that includes the physical, mental and social areas. Along these lines, the concept of health-related physical fitness (HRPF) has emerged as an important marker of health, and it is usually defined as a multidimensional construct containing the components cardiorespiratory endurance, muscular strength, muscular endurance, flexibility and body composition [9]. In this regard, it is worth highlighting the position that the state of health is the product of both internal and external factors [10]. Going further, Prince et cols. [11] asserted that there is no health without mental health. This implies taking into account mental fitness, which involves a dynamic process of individual psychological self-regulation resulting from the interaction between a person and their changing environment [12]. Following the theory of change, we would have to consider when approaching fitness how certain processes lead to certain results that make up the final state [13]. Therefore, to be able to affect or modify the result, we must understand and manage the involved psychological processes.

Regarding the practicing of physical activity and sports, certain psychological, sociodemographic and psychosocial variables have been reported as moderators. This is the case for age and sex, as there is a general decrease in sports practice, especially in older age [14,15], this being especially accentuated in the case of females, who show lower fitness and activity levels and greater abandonment [16,17]. Indeed, adolescence is a critical period given that important psychosocial changes take place, such as the development of the self-concept [18], from both physical and psychological perspectives [19], affecting their psychological well-being [20]. This implies the need to delve into factors that appear in the early stages of development, especially those that are not usually considered as psychological and social. In fact, even the acquisition of motor abilities, which follows genetic patterns related to the motor brain areas and the nervous system, is influenced by the social environment and one’s personality [21]. However, it is in childhood that habits and conceptions are established that will mark the life of the person.

In order to prevent and manage expectations and motivation to avoid both abandoning the practice and attain a protective factor for physical and psychological health, it will be important to understand the relationship between the perceived levels of fitness and the desired ones—that is, the attitude towards fitness given a certain level.

Self-perception refers to the way children define themselves and think about the way they are recognized by others [22]. Currently, health policies are aimed at getting children to increase their fitness levels through the performance of physical activity [23]. However, scant research has focused on whether children believe that their fitness levels need to be improved or whether they wish to have higher HRPF. This is an important aspect to consider, since it could determine the success of said health policies among children.

For all the above, in the present study we analyzed the relationship between the self-perceived and the desired health-related physical fitness in a sample of children.

## 2. Methods

### 2.1. Participants

Spanish children enrolled in primary education (age range 10–11 years) were invited to take part in this study through invitation letters, after contacting their physical education (PE) teacher. Inclusion criteria were: (a) to attend an urban school that has a collaboration agreement with the University of Vigo; and (b) to have standard physical and mental development. Exclusion criteria were: (a) to be exempted from PE lessons; and (b) to have medical problems that prevented the performance of the proposed tests. Out of the 336 children who volunteered to take part in the study, 330 of them completed all measurements. Informed written consent was requested and obtained from the parents and school headmasters. The study protocol was approved by the Ethics committee of the [BLINDED FOR REVIEW].

### 2.2. Measurements

#### 2.2.1. Self-Perceived and Desired Health-Related Physical Fitness (HRPF)

For assessing both self-perceived and desired HRPF at once, we used a slightly modified version of the self-perceived HRPF questionnaire for children (SPHQ-C). This questionnaire has shown accurate validity evidence based on internal structure, particularly good test–retest reliability and a fair convergent validity when administered to pre-pubertal children [24].

The SPHQ-C includes two sets of questions. There are five items asking how children perceive their levels of cardiorespiratory and muscular fitness, flexibility and body composition, according to a 1–5 rating scale (the higher the score, the better the fitness self-perception, except for the body composition items that are rated 1–3–5–3–1). A second set of questions ask the children to report how fit they think they are in comparison with other children of the same age. A total score ranging from 9 to 45 points is obtained after the SPHQ-C’s administration.

For the purpose of this research, in each of the SPHQ-C items, apart from asking the children to choose the statement that came closest to their self-perceived HRPF level (i.e., I see myself as…), we added a second question asking them to choose the statement that came closest to the HRPF level they would like to have (i.e., I would like to be…). This was performed following a previous procedure for identifying and comparing respondents’ perceptions of current and preferred capabilities [25]. This second line of questions also resulted in a score ranging from 9 to 45 points.

#### 2.2.2. Health-Related Physical Fitness

The children’s height (cm) and weight (kg) were registered by means of a statimeter and a digital scale. The obtained data were used to identify body mass index (BMI), an indicator of body composition, by dividing the weight by the height (kg/m^2^). Cardiorespiratory fitness, muscular strength and muscular endurance were assessed through three field-based tests (Course-Navette, hand-held dynamometry and sit-ups) included in the Eurofit battery [26]. The V-sit test [27] was chosen for assessing flexibility levels.

### 2.3. Procedures

A four-week period was used to collect the data. Each participant answered the questionnaire twice while in class with a 15-day interval in between. This procedure provided information regarding the relative (test–retest) reliability of the modified version of the SPHQ-C. One week after the second administration of the modified SPHQ-C, the children performed the field-based test, including the anthropometric assessment (last week of the four-week schedule). Four research assistants (all fourth-year university PE students) administered all questionnaires and fitness tests under the direction of the main investigator of the study.

### 2.4. Statistical Analysis 

The internal consistency and the test–retest reliability of the modified version of the SPHQ-C were identified by means of the Cronbach’s α and the intraclass correlation coefficient (ICC), respectively. A descriptive analysis of the global and partial scores related to both the self-perceived and the desired HRPF was carried out. For this purpose, the questions were grouped into four sections as follows: muscular strength (Q1–Q3), cardiovascular fitness (Q4–Q5), flexibility (Q6–Q7) and body composition (Q8–Q9). Since very few children wished to have low fitness levels when asked about their desired HRPF, the categories “very poor,” “poor” and “average” were grouped and scored together as one. Gender differences in the distribution of the global and by-section results for both questionnaires were analyzed using a Chi-squared test. Shapiro–Wilk tests were performed to assess normality. Differences between the global and partial scores related to both self-perceived and desired HRPF levels were analyzed by performing first a Kruskal–Wallis test, and then a post hoc Mann–Whitney test. In order to explore the existence of a relationship between the desired and the objectively assessed HRPF, associations between the partial scores obtained in the desired HRPF questions and the scores obtained in the respective field-based tests were identified by means of Kruskal–Wallis test. In these analyses, body composition data were interpreted as follows: 1 (very thin), 2 (thin), 3 (normal weight), 4 (overweight) and 5 (obese) for clarification purposes. Data were analyzed using Stata.

## 3. Results

The final sample was made up of 148 girls (mean age: 10.78 ± 0.67; BMI: 19.80 ± 3.70) and 182 boys (mean age: 10.82 ± 0.61; BMI: 19.45 ± 2.85). The questions regarding desired HRPF showed moderate internal consistency (Cronbach’s α: 0.671) and good test–retest reliability (ICC: 0.761).

The mean global score regarding self-perceived HRPF was lower than that observed for desired HRPF (31.5 ± 4.4, 62.5% of the score range vs. 40.0 ± 3.9, 86% of the score range). These results indicate that in general, children shared a desire to improve their self-perceived HRPF.

Table 1 shows the distribution for both boys and girls of the total and partial scores of the SPHQ-C. Regarding self-perceived HRPF, significant differences were observed for muscular and cardiovascular fitness, favoring boys, and for flexibility, favoring girls. A similar trend was observed for desired HRPF, although in this case girls showed a significant and greater desire for having a normal weight.

The degree of association between the objectively assessed and the desired HRPF is shown in Table 2. A significant relationship between the scores obtained in the hand-held dynamometer test and the desire for being stronger was observed among boys and girls.

Significant associations between the scores obtained in the “Course-Navette” and in the “V-Sit and Reach” tests and the desire for having greater cardiorespiratory fitness and flexibility were also observed among girls, respectively. According to the obtained data, the boys who already had good muscular strength levels, and the girls who showed high muscular, cardiovascular and flexibility fitness levels in the field-based tests, were the ones who wished to have greater HRPF.

**Figure 1:**
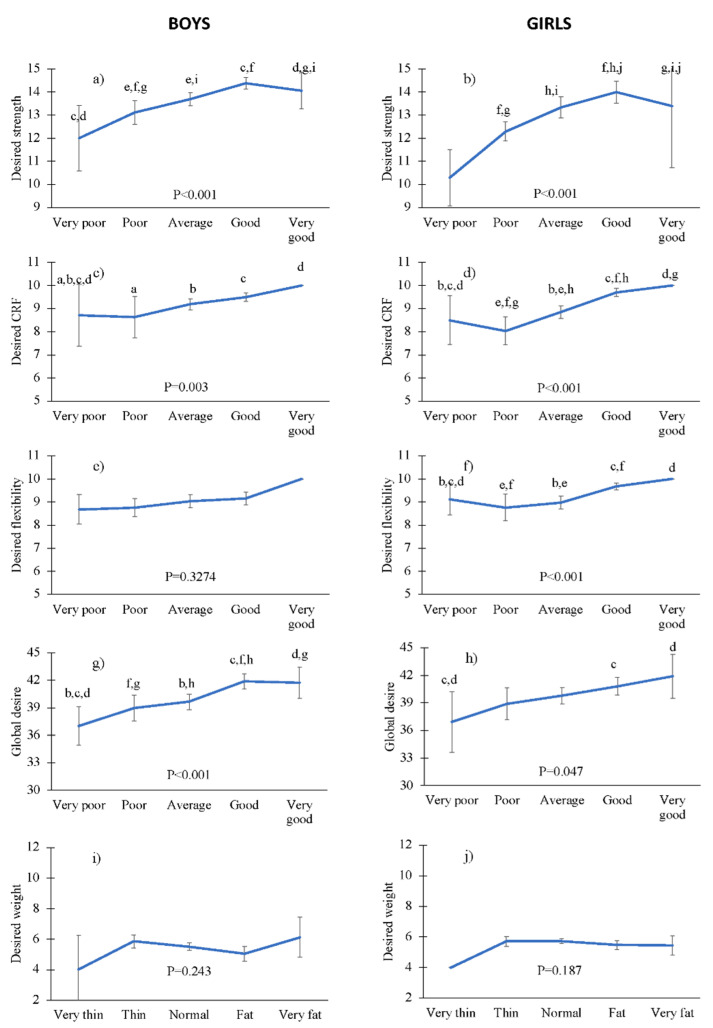
Associations between the perceived and the desired fitness, stratified by boys/girls (graphs (**a**) through (**j**)). Lowercase letters indicate significant differences (*p* < 0.05) in post hoc Mann–Whitney test comparison between pairs: (**a**) very poor vs. poor; (**b**) very poor vs. average; (**c**) very poor vs. good; (**d**) very poor vs. very good; (**e**) poor vs. average; (**f**) poor vs. good; (**g**) poor vs. very good; (**h**) average vs. good; (**i**) average vs. very good; (**j**) good vs. very good.

The relationship between how the children perceived their HRPF levels and how they would like them to be can be seen in Figure 1. In general, all children wished to have higher HRPF levels. Significant differences were observed among the children considering the observed SPHQ-C mean scores, indicating that those who obtained higher self-perceived HRPF scores were the ones who showed the strongest desire for achieving higher HRPF levels.

## 4. Discussion

This study aimed to assess the relationship between the self-perceived and desired HRPF in a sample of children. Our results could be of interest for those in charge of designing public health policies aimed at increasing the children’s fitness levels through the promotion of physical activity.

After testing the HRPF of our sample, we found that boys showed higher levels of muscular and cardiorespiratory fitness, whereas girls performed substantially better on the flexibility test. These differences have also been previously reported in other studies [4] and may be due to both morpho-functional and socio-cultural factors [28,29], leading to less frequent and less intense physical activity among girls [30].

Regarding the desire to improve the HRPF, we found that the participants with better measured HPRF showed a greater desire to improve their HRPF levels. Specifically, our data showed associations between assessed hand grip strength and the desire for being stronger among boys and girls, and the assessed cardiorespiratory fitness and flexibility and the desire for improving these components among girls. We also found that girls have a significantly higher desire to have a normal weight. This finding is in line with previous observations indicating that a considerable number of adolescents girls perceive themselves as being overweight, in comparison with boys [31]. This fact could be due to the influence of family and peer groups as primary socializers, and media or social values [32,33]. Thus, although it may be striking that this is present at around 10 years of age, it has already been shown that the weight gender stereotypes begin at 3 years of age (ranging from 3 to 7) [34]. The eradication of this social sexism is thus fundamental for the well-being and development of children, especially in adolescent girls [35]. Similarly to the results of measured and desired HRPF, we found that those who reported higher self-perceived scores also showed a stronger desire to improve their HRPF [36].

Altogether, our findings emphasize the relationship between motivation and behavior, highlighting the need to know people’s own perceptions to offer better support, given the importance of motivation, among other psychological variables, to increase physical activity and sports participation [37,38,39]. In fact, previous studies indicate that convincing children of the need to be more active is a key aspect to promoting changes in physical activity levels [40]. It is therefore important to identify which children are not already convinced, so that tailored, theoretically informed interventions can be developed. In addition, children’s self-perceptions of other related constructs, such as their motor skills, are also important to be considered. Previous studies have reported that the self-perception of low motor skills, and having actual poor motor competence, have been linked to lower HRPF and lower physical activity levels in children and adolescents [41,42,43]. It is therefore necessary that public health policies and interventions are also directly oriented towards the development and improvement of motor skills, particularly at young ages.

Another important aspect of identifying children with lower levels of self-reported HRPF is that it could also be useful to spot those children who are more prone to the abandonment of physical activity participation. Indeed, it has been suggested that in children, low self-reported physical fitness could lead to a reduction in physical activity [44]. This is particularly important in the case of girls, since they have higher rates of abandonment, especially during adolescence [16,17]. We must not forget, however, other contextual or socioeconomic variables that also condition the levels of physical activity, such as parental health status, lack of facilities to participate in exercise programs or previous experiences within the practice of physical activity [45].

Despite the originality of this research, there are certain limitations that should be addressed. First, the sample size was small and specifically selected from Spanish urban schools, limiting the generalizability of our findings. Second, we did not assess the validity of the items added to the SPHQ-C. Finally, cardiorespiratory fitness and flexibility levels were not measured through gold standard tests, a procedure that would have yielded more solid results.

## 5. Conclusions

In a sample of Spanish primary education students, we found that children generally shared a desire to improve their self-perceived HRPF. Those who perceived themselves as already being fit were the ones who showed the strongest desire for achieving higher HRPF levels. Health policies aimed at improving the fitness levels of children should be specially targeted at those who are less fit, since they are the least inclined to change their fitness levels.

## Figures and Tables

**Table 1:** Distribution for both boys and girls of the total and partial scores of the SPHQ-C.

Dimension	Rating	Boys	Girls	*p*-Value
Perceived strength	Very poor	7 (3.85%)	17 (11.49%)	0.000
	Poor	38 (20.88%)	61 (41.22%)	
	Average	74 (40.66%)	45 (30.41%)	
	Good	47 (25.82%)	20 (13.51%)	
	Very good	16 (8.79%)	5 (3.38%)	
Perceived endurance	Very poor	7 (3.85%)	12 (8.11%)	0.002
	Poor	16 (8.79%)	27 (18.24%)	
	Average	69 (37.91%)	65 (43.92%)	
	Good	74 (40.66%)	37 (25%)	
	Very good	16 (8.79%)	7 (4.73%)	
Perceived flexibility	Very poor	22 (12.09%)	8 (5.41%)	0.001
	Poor	47 (25.82%)	21 (14.19%)	
	Average	65 (35.71%)	55 (37.16%)	
	Good	45 (24.73%)	53 (35.81%)	
	Very good	3 (1.65%)	11 (7.43%)	
Global perception	Very poor	11 (6.04%)	12 (8.11%)	0.372
	Poor	21 (11.54%)	27 (18.24%)	
	Average	85 (46.7%)	60 (40.54%)	
	Good	50 (27.47%)	40 (27.03%)	
	Very good	15 (8.24%)	9 (6.08%)	
Perceived weight	Very thin	3 (1.65%)	1 (0.68%)	0.377
	Thin	30 (16.48%)	21 (14.19%)	
	Normal	94 (51.65%)	86 (58.11%)	
	Fat	47 (25.82%)	29 (19.59%)	
	Very fat	8 (4.40%)	11 (7.43%)	
Desired strength	Very poor-Average	14 (7.69%)	39 (26.35%)	0.000
	Good	57 (31.32%)	52 (35.14%)	
	Very good	111 (60.99%)	57 (38.51%)	
Desired endurance	Very poor-Average	11 (6.04%)	25 (16.89%)	0.002
	Good	59 (32.42%)	53 (35.81%)	
	Very good	112 (61.54%)	70 (47.3%)	
Desired flexibility	Very poor-Average	24 (13.19%)	10 (6.76%)	0.037
	Good	74 (40.66%)	51 (34.46%)	
	Very good	84 (46.15%)	87 (58.78%)	
Global desire	Very poor-Average	9 (4.95%)	11 (7.43%)	0.601
	Good	37 (20.33%)	27 (18.24%)	
	Very good	136 (74.73%)	110 (74.32%)	
Desired weight	Very thin	21 (11.54%)	2 (1.35%)	0.001
	Thin	40 (21.98%)	41 (27.7%)	
	Normal	114 (62.64%)	107 (72.3%)	
	Fat	4 (2.20%)	0 (0%)	
	Very fat	3 (1.65%)	0 (0%)	

**Table 2:** Associations between the objectively assessed and desired fitness.

Measured Fitness (Tool, Units)	Desired Fitness	Boys			Girls		
		*n*	Mean (SD)	*p*-Value	*n*	Mean (SD)	*p*-Value
Strength (dynamometer, kg)	Very poor-Average	14	16.81 (6.14)	0.008	39	16.00 (5.47)	0.004
	Good	57	16.40 (4.55)	C	52	16.90 (4.58)	b, c
	Very good	111	18.73 (4.68)		57	19.67 (5.68)	
Strength (sit-ups, number)	Very poor-Average	14	19.86 (4.79)	0.799	39	17.26 (7.35)	0.3134
	Good	57	19.77 (5.96)		52	18.35 (5.37)	
	Very good	111	20.56 (5.22)		57	19.35 (5.46)	
Cardiorrespiratory fitness (Course-Navette)	Very poor-Average	11	3.59 (2.35)	0.323	25	2.26 (1.02)	0.002
	Good	59	4.62 (2.76)		53	3.73 (2.68)	a, b
	Very good	112	4.77 (2.81)		70	3.79 (2.04)	
Flexibility (V-Sit and Reach, cm)	Very poor-Average	24	11.33 (7.98)	0.451	10	15.40 (7.18)	0.001
	Good	74	13.80 (8.77)		51	19.25 (9.87)	b, c
	Very good	84	14.08 (9.10)		87	24.74 (10.86)	
Body Mass Index (kg/m^2^)	Very thin	21	19.81 (2.40)	0.719	2	24.02 (10.70)	0.676
	Thin	40	19.77 (3.00)		41	19.35 (3.56)	
	Normal weight	114	19.24 (2.92)		107	19.89 (3.60)	
	Fat	4	19.84 (2.48)		0	-	
	Very fat	3	19.79 (2.32)		0	-	

Lowercase letters indicate significant differences (*p* < 0.05) in post hoc Mann–Whitney test comparison between pairs (a: very poor–average vs. good; b: very poor–average vs. very good; c: good vs. very good).

## Data Availability

Data is available upon request addressed to the contact author.
